# Histopathological Findings in an Unclassifiable Case of Empty Nose Syndrome with Long-term Follow-up

**DOI:** 10.7759/cureus.2655

**Published:** 2018-05-20

**Authors:** Deyan L Dzhenkov, George S Stoyanov, Radoslav Georgiev, Nikolay Sapundzhiev

**Affiliations:** 1 Department of General and Clinical Pathology, Forensic Medicine and Deontology, Faculty of Medicine, Medical University – Varna “Prof. Dr. Paraskev Stoyanov”, Varna, BGR; 2 Department of General and Clinical Pathology, Forensic Medicine and Deontology, Medical University – Varna "Prof. Dr. Paraskev Stoyanov", Varna, BGR; 3 Department of Radiology and Radiotherapy, Division of Radiology, Faculty of Medicine, Medical University Varna "Prof. Dr. Paraskev Stoyanov", Varna, BGR; 4 Department of Neurosurgery and Ent, Division of Ent, Faculty of Medicine, Medical University Varna "Prof. Dr. Paraskev Stoyanov", Varna, BGR

**Keywords:** empty nose syndrome, pathology, histology, nasal epithelium, case report

## Abstract

One of the major components of the functional process in the nasal cavity is taken up by the respiratory epithelium covering the posterior two-thirds of the nasal cavity. Disruption in the cytoarchitectonics and subcellular changes in this epithelium results in a number of functional changes in the nasal cavity. One of the rare and usually iatrogenic disturbances of this type is described in 1996, although noticed and discussed significantly earlier, by Kern and Stenkvist empty nose syndrome (ENS) or secondary atrophic rhinitis. The clinical hallmarks of ENS are described as paradoxical feeling for nasal obstruction in the presence of actually widened nasal airways. This phenomenon is attributed to the efferent neuronal signal dissociation accompanying the changes in the nasal mucosa. Herein we report the findings in a 50-year-old male. The patient presented with chronic right-sided headache, foul discharge and complaints of a stuffed nose in 2011. Endoscopy and computed tomography (CT) showed complete destruction of the hard plane, nasal septum, and right maxillary septum, leading to a formation of a huge neocavity. Due to the past medical history and the severity of the case biopsy specimens were obtained under general anesthesia. The sections showed severe but unspecific changes of the nasal epithelium with areas of minimal remaining preserved respiratory epithelium. Based on the clinical data and endoscopic, CT and histomorphologic data, despite the case is not applicable to the current classification of ENS, the diagnosis of ENS was accepted based on the combined extensive but unspecific findings. A seven-year follow-up period included multiple hospital admissions for monitoring of the condition and extensive sinus lavage. No advancement was noticed.

## Introduction

One of the major components of the functional process in the nasal cavity is taken up by the respiratory epithelium covering the posterior two-thirds of the nasal cavity [1]. Disruption in the cytoarchitectonics and subcellular changes in this epithelium results in a number of functional changes in the nasal cavity ranging from allergic reactions to inflammation and carcinogenesis [2]. These conditions vary wildly in pathogenesis raging from idiopathic to inflammatory and iatrogenic [3].

One of the rare and usually iatrogenic disturbances of this type is described in 1996, although noticed and discussed significantly earlier, by Kern and Stenkvist empty nose syndrome (ENS) or secondary atrophic rhinitis [4]. ENS is a condition where the respiratory epithelium is widely replaced by stratified epithelium, often keratinizing, as a result of excessive damage to the nasal cavity from repeated endonasal procedures, resulting in predominantly functional changes [1,3,5].

The clinical hallmarks of ENS are described as paradoxical feeling for nasal obstruction in the presence of actually widened nasal airways [6]. This phenomenon is attributed to the efferent neuronal signal dissociation accompanying the changes in the nasal mucosa. Other more unspecific symptoms include nasal cavity dryness, recurrent suppurative infections with foul discharge and crustation [1,2].

The most common reasons for ENS as already mentioned are repeated endonasal procedures, based on the locations of which ENS can be classified into four categories: ENS due to inferior turbinate, middle turbinate, and combined inferior and middle turbinate resections, and recurrent turbinate sparing procedures [4].

## Case presentation

Herein we report the findings in a 50-year-old male. The patient presented with chronic right-sided headache, foul discharge and complaints of a stuffed nose in 2011. Previous medical history included maxillary osteomyelitis in 2009 with multiple interventions resulting in a complex mediofacial resection, with a subtotal defect of the hard plate, maxillary sinus, and nasal septum. Concomitant diseases included depression and type two diabetes mellitus on peroral therapy.

**Figure 1:**
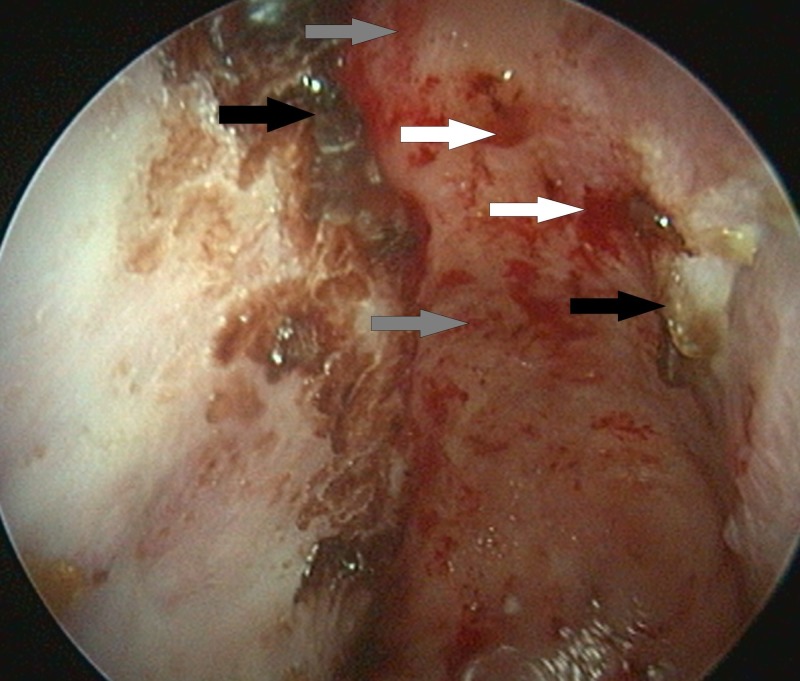
Endoscopic view of the nasal cavity. There is a complete lack of nasal septum with severe epithelial changes – various crustations (black arrows), hemorrhage (white arrows) and ulcerations (grey arrows).

**Figure 2:**
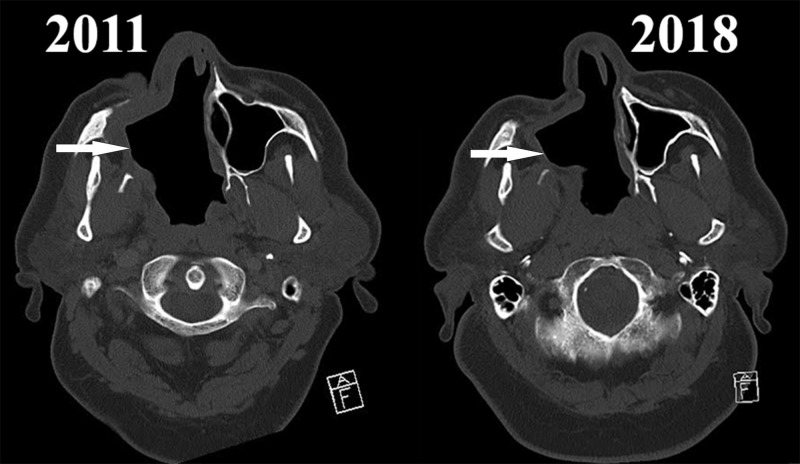
H&N CT scans from 2011 to 2018 – the neocavity (arrows) shows no significant changes. H&N: Head and neck; CT: Computed tomography

A nasal endoscopy and computed tomography (CT) showed a huge pathologic cavity resulting from communication between the oral cavity, right maxillary sinus, and nasal cavity, with a minor communication with the right orbit (Figures 1-2).

Due to the past medical history and the severity of the case biopsy specimens were obtained under general anesthesia. The tissues were fixated in 10% buffered formaldehyde solution and later embedded in paraffin (FFPE) for staining with hematoxylin and eosin (H&E) and Azan.

**Figure 3:**
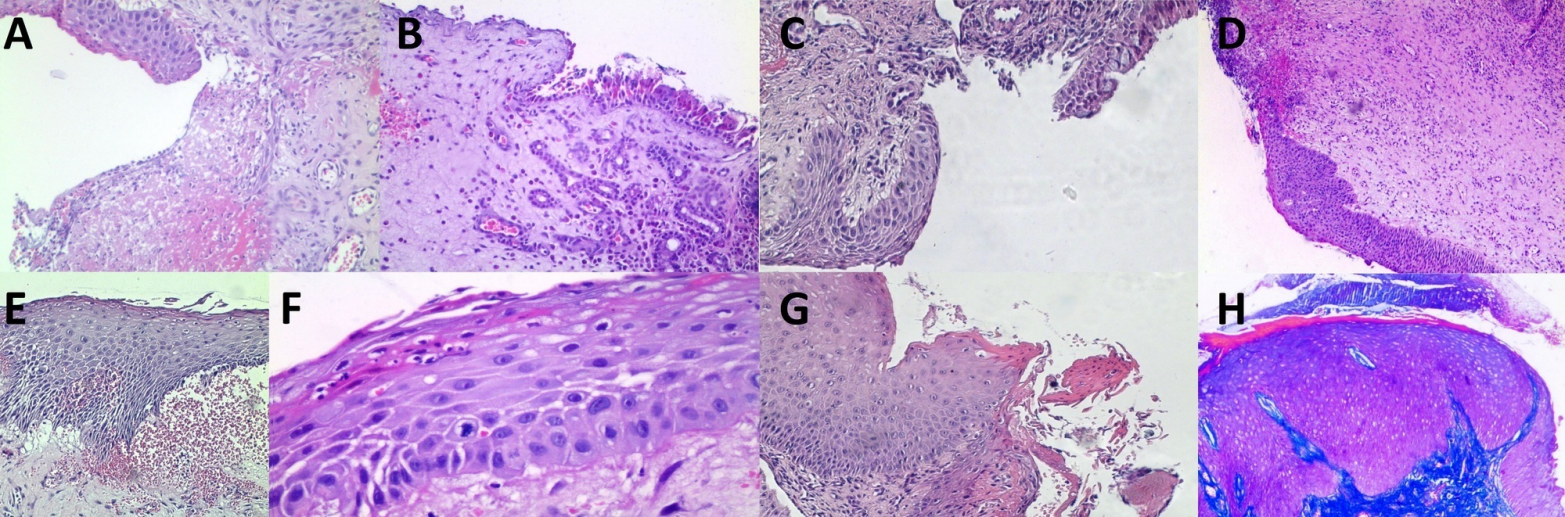
Spectrum of histological changes in the mucosal lining. (A) Squamous cell metaplasia and adjacent erosion and fibrino-purolent exudate, H&E, original magnification 200x; (B) Erosions adjacent to remaining respiratory epithelium, H&E, original magnification 200x; (C) Squamous cell metaplasia with adjacent preserved respiratory epithelium with goblet cells, H&E, original magnification 200x; (D) Squamous cell metaplasia with ulceration and submucosal granulation tissue formation, H&E, original magnification 40x; (E) Squamous cell metaplasia with keratinization, H&E, original magnification 200x; (F) Squamous cell metaplasia with keratinization, H&E, original magnification 400x; (G) Squamous cell metaplasia with abundant keratinization adjacent to erosion, H&E, original magnification 200x; (H) Squamous cell metaplasia with keratinization and acanthosis with severe submucosal scar tissue formation, Azan, original magnification 200x. H&E: Hematoxylin and eosin

**Figure 4:**
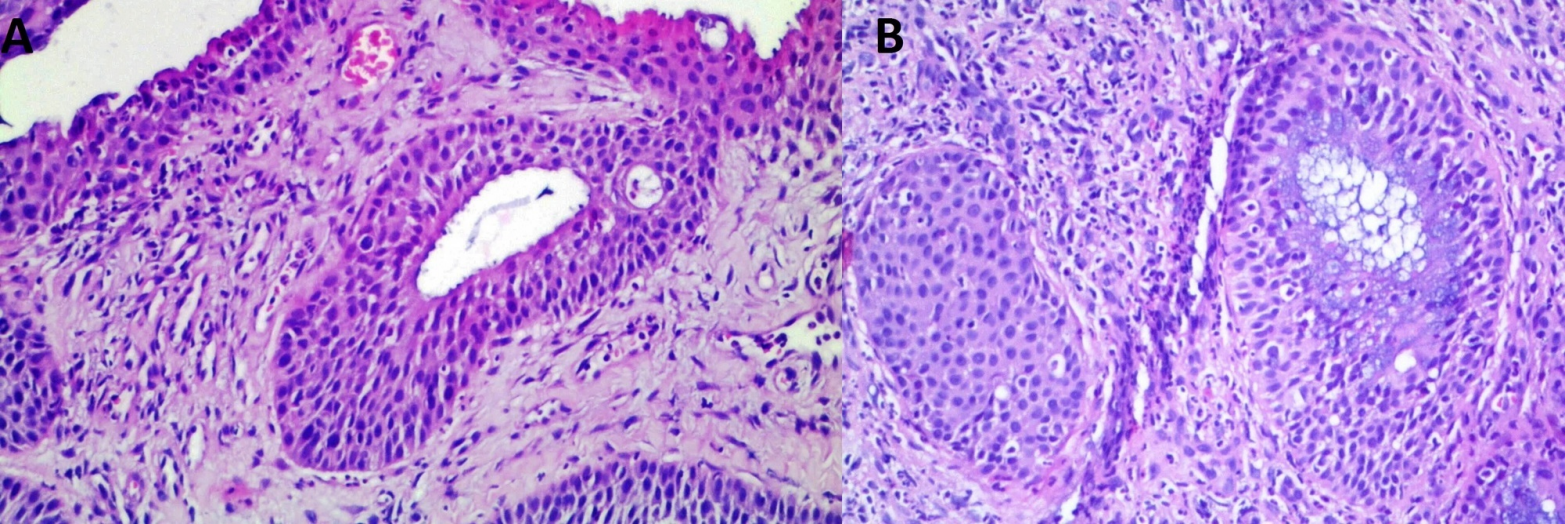
Spectrum of histological glandular changes. (A) Squamous cell metaplasia in submucosal gland duct, H&E, original magnification 200x; (B) Complete replacement of submucosal glands with squamous cells, forming submucosal nest without signs of atypism, H&E, original magnification 200x. H&E: Hematoxylin and eosin

**Figure 5:**
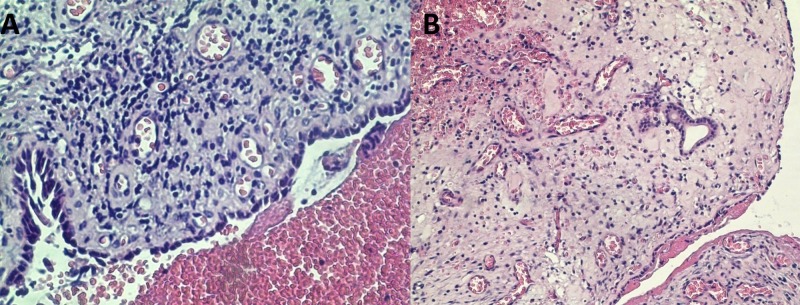
Spectrum of histological submucosal changes. (A) Granulation tissue with abundant plasmocytes, inflammatory degenerated covering epithelium and hemorrhage, H&E, original magnification 400x; (B) Granulation tissue with abundant inflammatory cells, H&E, original magnification 200x. H&E: Hematoxylin and eosin.

The sections showed severe but unspecific changes of the nasal epithelium with areas of minimal remaining preserved respiratory epithelium (Figure 3). These changes included zones of reserve cell hyperplasia, epithelial erosions, and ulcers, squamous cell metaplasia with acanthosis and zones of abundant keratinization (Figure 3). Evident unspecific changes were also present in the submucosa with squamous cell metaplasia of the submucosal glands in some cases leading to complete replacement of the glandular epithelia and resulting in submucosal squamous cell nests, without signs of cellular atypia (Figure 4). The interstitium was also severely affected by abundant zones of granulation tissue, fibrosis, and signs of chronic inflammatory infiltrate with lymphocytes and plasma cells, however giant cells were absent (Figure 5). The adjacent bones showed no signs of active inflammation.

 

Based on the clinical data and the endoscopic, radiologic and histomorphologic data, despite the case is not applicable to the current classification of ENS, the diagnosis of ENS was accepted based on the combined extensive but unspecific findings. Due to the current classification criteria, however, the case was concluded to be ENS not elsewhere classified (NEC) as the currently used classification does not include such an entity.

The patient was discharged following training for self-lavage of the neocavity, due to refusal for obturator placement to compartmentalize the neocavity and restore physiological relation of its sections. A seven-year follow-up period included multiple hospital admissions for monitoring of the condition and extensive sinus lavage. No advancement was noticed on endoscopy and CT. No further histological evaluations were performed due to the steady clinical course and lack of evidence for malignant transformation of the now squamous epithelial lining of the neocavity.

## Discussion

Many current revisions of classifications are now expanding to include NEC types, due to the wide variety of clinical, endoscopic, radiological and histological criteria [4]. The current case is evidence of the need for such entries as it does not follow the criteria, whilst still being a typical case of ENS, attributable to multiple previous medical interventions. The severity of the condition, however, together with the collected data is evidence of the need for classification into the ENS clinical spectrum.

The current case, despite its severity, shows that continuous monitoring and patient education can lead to a relatively stable condition, without progression over a seven-year follow-up period, despite initial presentation.

## Conclusions

The current report illustrates the wide range of histological changes found in a single ENS case. Despite being a secondary iatrogenic entry, ENS classifications should widen the scope to include histological criteria, as the severe changes illustrated by this case, can be considered as a precancerosis.
